# *In situ* growth of PEDOT/graphene oxide nanostructures with enhanced electrochromic performance

**DOI:** 10.1039/c8ra01153b

**Published:** 2018-04-12

**Authors:** Yingdi Shi, Yong Zhang, Kai Tang, Yanbin Song, Jiewu Cui, Xia Shu, Yan Wang, Jiaqin Liu, Yucheng Wu

**Affiliations:** School of Materials Science and Engineering, Hefei University of Technology Hefei 230009 China zhangyong.mse@hfut.edu.cn ycwu@hfut.edu.cn; Key Laboratory of Advanced Functional Materials and Devices of Anhui Province Hefei 230009 China; Institute of Industry & Equipment Technology, Hefei University of Technology No. 193 Tunxi Road Hefei Anhui 230009 China

## Abstract

Poly(3,4-ethylenedioxythiophene) (PEDOT)/graphene oxide (GO) hybrid nanostructures have been obtained by an *in situ* electro-polymerization process. Field emission scanning electron microscope observation indicates that the hybrid nanostructures consist of uniform and well-dispersed PEDOT nanoparticles integrated on the networked GO nanosheets. Surface chemistry and structure of the hybrid nanostructures have been characterized by X-ray photoelectron spectroscopy (XPS) and Raman spectroscopy. Electrochemical and optical property measurements demonstrate that the hybrid nanostructures exhibit significantly improved electrochromic performance compared with the pristine PEDOT nanostructure. The contrast between coloring and bleaching state of the PEDOT nanostructure at 480 nm increases from 23.4% to 31.4% after hybridizing with GO nanosheets. The coloring time and bleaching time are shortened from 1800 ms to 300 ms and 1500 ms to 400 ms, respectively, while the coloring efficiency increases from 53.5 cm^2^ C^−1^ to 64.9 cm^2^ C^−1^ after the hybridization. The obtained PEDOT/GO hybrid nanostructures promise great potential in developing novel electrochromic materials for smart windows and other energy saving applications.

## Abstract 2

Designed growth of a novel PEDOT/graphene oxide (GO) hybrid and obtained hybrid demonstrates superior electrochromic performance.

## Graphical abstract

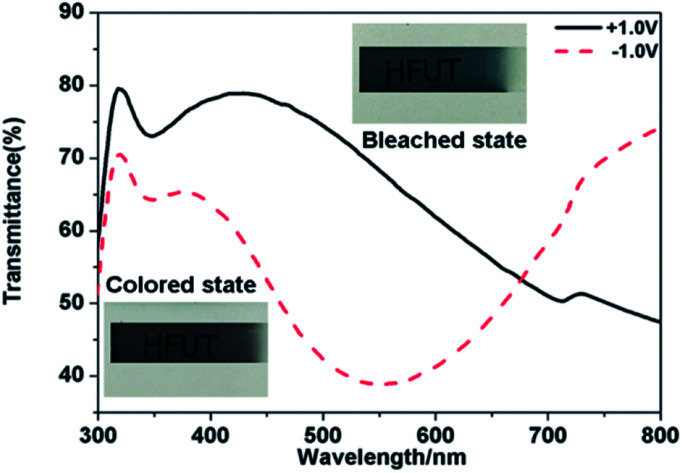

## 1 Introduction

Electrochromic materials have attracted steadily increasing interest owing to their distinct function of exhibiting reversible modulation in optical transmittance, absorbance or reflection consuming low electric power. The different characteristics of absorption-type, transmission-type and reflective-type electrochromic devices (ECDs) render them ideal candidates for energy-saving windows, smart displays, electronic papers, indoor safety as well as military camouflage.^1,2^ Compared to inorganic electrochromic materials, conjugated polymers have the advantages of fast response speed, multicolor changes, easy design and cost effectiveness.^3,4^ Poly(3,4-ethylenedio-xythiophene), or PEDOT, is a typical cathodically coloring conjugated polymer featuring high color efficiency, high electrical conductivity, high transmissivity for visible radiation, short response time and resistance to degradation in the doped form.^5–8^ Previous reports indicated that the electrochromic performance of PEDOT can be obviously improved by controlling the size of the PEDOT in nanoscale, and various low dimensional PEDOT nanostructures have been developed such as PEDOT nanoparticles,^9^ nanowires,^10^ nanotubes^11,12^ and nanobelts.^13,14^ Nevertheless, optical contrast and recycling stability of PEDOT are still not high enough to meet the requirement for practical applications.

Utilizing designed monomers or combining different kinds of monomers to produce electrochromic materials has been considered as a promising approach for ECDs development and significant improvement has been achieved.^15,16^ On the other hand, hybridization between organic and inorganic components in nanoscale has recently been proposed as an alternative solution for developing novel ECDs.^17^ Fortunately, PEDOT has a π-conjugated skeleton which can incorporate organic and/or inorganic molecules to achieve hybrids with enhanced or modulated electrochromic properties. Recently, different carbon materials such as graphene, carbon nanotubes and fullerenes have been hybridized with conjugated polymers for various practical applications such as dye-sensitized solar cells, energy storage systems, biological or physical sensors and biomedical applications, in which graphene oxide nanosheets exhibit especially favorable performance owing to their high specific surface area and superior dispersion in many common solvents.^18–21^ In addition, rich oxygenic functional groups on the GO nanosheets can^22,23^ make the surface negatively charged, which facilitates the electrical polymerization of the conductive monomer on the GO surface. Moreover, outstanding flexibility of GO nanosheets renders it an ideal framework for buffering volume changes of the electrode during the electrochromic process.^24^ It is expected that the growth of organic–inorganic hybrids could combine the advantages between the two and the confined growth of the organic materials in nanoscale may promise enhanced electrochromic performance. However, the research on the electrochromic properties of PEDOT/GO hybrid nanostructures still remains lacking.

In this paper, we report a novel PEDOT/GO hybrid nanostructures by *in situ* electro-polymerization in which GO nanosheets are beaded with well-dispersed PEDOT nanoparticles without using any additional dopants. We combined transparent conducting electrode and electrochromic layer together to obtain transmission-type ECDs which can adjust the transmittance of light under the external electric field. Microstructure, surface chemistry and electrochromic properties of the hybrid nanostructures have been scrutinized. This type of the hybrid nanosheets are expected to possess high surface area that facilitate the electron transportation and ions injection/excitation in which GO can act as framework for buffering volume changes of the electrode during the electrochromic process.

## 2 Experimental

GO solution was prepared based on the modified Hummer's method. 0.1 M EDOT and 20 ml GO solution (5 mg ml^−1^) were mixed and dispersed in 20 ml ethanol and treated by ultra-sonication for 3 hours to be used as an electrolyte. *In situ* electro-polymerization was conducted on a piece of ITO coated glass with the immersing area of 2 × 0.5 cm^2^ in a three-electrode system for 10 s at 2.5 mA cm^−2^ using chronopotentiometry, in which a platinum wire and a Ag/AgCl electrode were used as counter and reference electrode, respectively. After the electro-polymerization, the sample was rinsed by ethanol for several times and dried in air. A pristine PEDOT film on an ITO coated glass was also prepared for comparison using the same parameters as those of growing hybrids except for that using 0.2 M LiClO_4_ to take the place of GO solution providing anions for the electro-polymerization process. Under the applied electric field, thiophene lost electrons to form free radicals on the electrode surface when the voltage reached the oxidation potential, and the dimer was further oxidized and synthesized into tetramer. The polythiophene with high degree of polymerization was finally formed. In order to maintain the electric neutral of the material, the cationic dopant would combine with the polythiophene film and the doped polythiophene film was finally obtained.

Graphite powder was purchased from Nanjing Xianfeng Nanomaterials Limited Company, China, LiClO_4_ powder (≥99%) was purchased from Sigma-Aldrich, EDOT monomer and absolute ethanol were purchased from Sinopharm. Morphology, composition, structure and optical properties of the samples were characterized by field emission scanning electron microscope (SU8020), X-ray diffractometer (CuKα radiation, *λ* = 0.15418 nm, Rigaku D/MAX 2500V), Confocal Micro-Raman spectrometer (Horiba, LabRAM HR Evolution), Thermo ESCALAB 250 X-ray photoelectron spectrometer, respectively. An electrochemical workstation (CHI760E) and UV-3600 spectrometer were used for electrochromic property evaluation.

In a typical process, a thin nanostructured electrochromic layer (PEDOT or PEDOT/GO layer) was assembled on a transparent conducting electrode (ITO glass) to form transmission-type ECDs. The electrochromic performance was evaluated by measuring the variation in the transmittance of light passing through the prepared ECDs. The measurement of the samples was carried out in a three-electrode electrochemical cell using 1 M LiClO_4_/PC solution as an electrolyte by combining a Shimadzu UV-3600 UV-VIS-NIR spectrophotometer and a CHI760E electrochemical workstation. The three-electrode system (the electrochromic film on ITO with the size of 2 × 0.5 cm, a platinum wire and a Ag/AgCl electrode were served as the working electrode, counter electrode and reference electrode, respectively) was inserted vertically into the cuvette filled with electrolyte. The step voltages of the PEDOT film and the PEDOT/GO hybrid film were set between −1.0 V and 1.0 V at a time step of 10 s, and the dynamic optical transmittance of the PEDOT film and the PEDOT/GO film was recorded at 480 nm.

## 3 Results and discussion

### 3.1 Microstructure

**Fig. 1:**
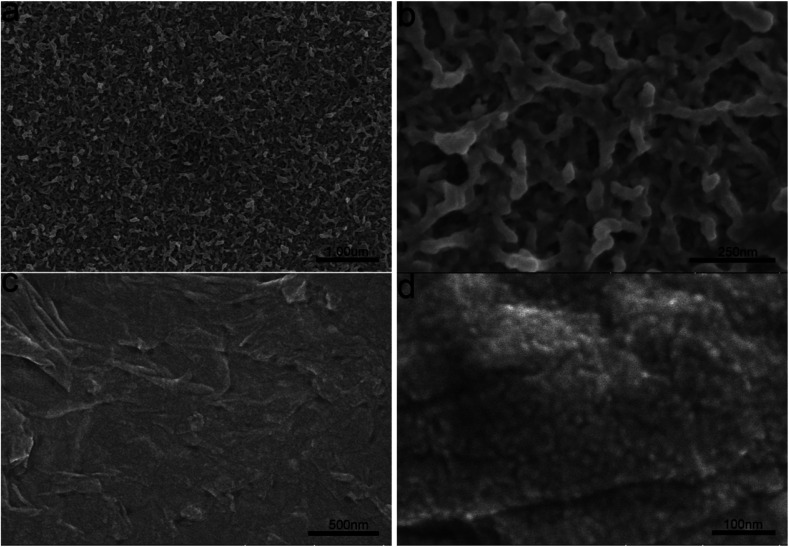
SEM images of (a) and (b) PEDOT film (c) and (d) PEDOT–GO film.

PEDOT/GO hybrid nanostructures were prepared by *in situ* polymerization of EDOT with GO nanosheets onto the ITO glass. For comparison, a pristine PEDOT film was also prepared on the ITO glass keeping the growth parameters unchanged. Fig. 1(a) shows a low magnification FESEM image of the pristine PEDOT film. High magnification FESEM image as shown in Fig. 1(b) indicates porous structure of the PEDOT film, which consists of interlinked nanorods with the diameters in the range of 20–60 nm. With the involvement of GO nanosheets into the growth, a PEDOT/GO hybrid film was obtained as shown in Fig. 1(c). Evidently, surface morphology of the hybrid film is quite different from that of the pristine PEDOT film. A close-up view of the hybrid film as shown in Fig. 1(d) suggests that the graphene oxide nanosheets are beaded with uniform and well-dispersed PEDOT nanoparticles. Typical size of the nanoparticles is in the range of 15–20 nm, forming a compact and rough surface with high surface area. The cause of the morphology difference between the hybrid nanostructures and the pristine PEDOT nanostructures can be ascribed to different interactions during the two electro-polymerization processes. While the growth of the pristine PEDOT nanostructures was dominated by the interaction between EDOT and small ions (*e.g.* ClO_4_^−^), the formation of the uniform PEDOT nanoparticles beading on the GO nanosheets could be attributed to the interaction between EDOT and functional groups on the GO nanosheets surface.^22^ With the involvement of GO into the growth, GO surface with functional groups served as a substrate favoring confined deposition of PEDOT nanoparticle with tiny size and uniform distribution.

### 3.2 Raman spectra

**Fig. 2:**
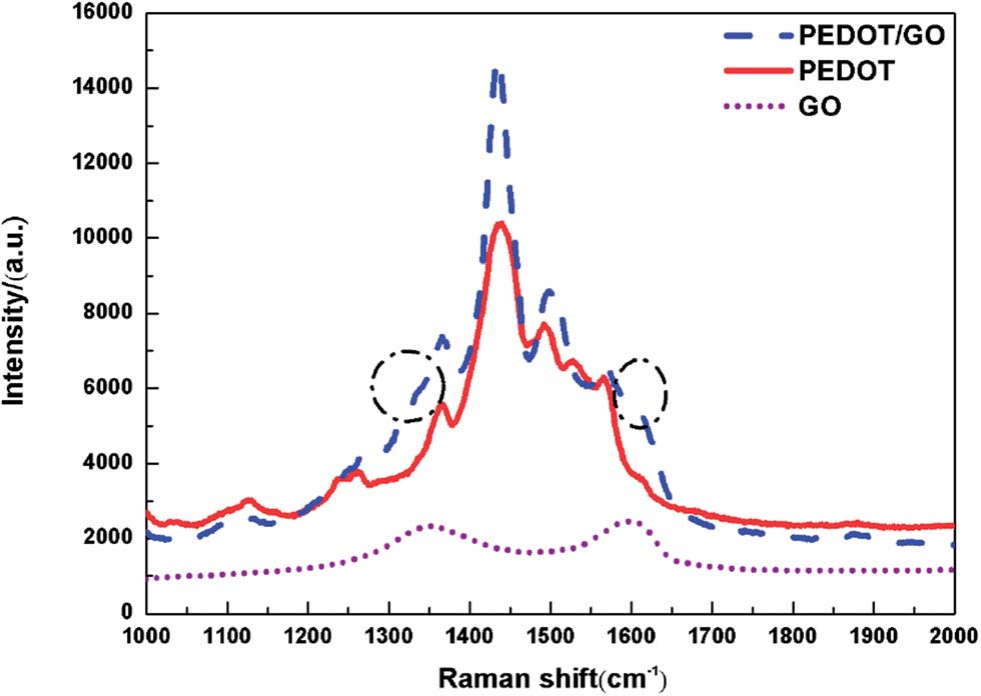
Raman spectra of pristine GO powders, PEDOT film and PEDOT–GO film.

Raman spectroscopy was utilized to characterize the hybrid nanostructures as previous reports.^25^Fig. 2 shows Raman spectra of GO nanosheets, the pristine PEDOT film and PEDOT/GO hybrid nanostructures, respectively. Raman spectrum of the GO nanosheets shows two bands centered at 1596 and 1353 cm^−1^, which can be attributed to well-documented G band and D band, respectively.^26^ Two strong peaks centered at 1436 and 1502 cm^−1^ for the pristine PEDOT film can be assigned to the asymmetric and symmetric C=C stretch. The bands at 1366 cm^−1^, 1267 cm^−1^ and 991 cm^−1^ originate from C_β_–C_β_ stretch, C_α_–C_α′_ inter-ring stretch and oxoethylene ring deformation, respectively,^27^ demonstrating characteristic peaks of PEDOT. In terms of Raman spectrum of PEDOT/GO hybrid nanostructures, two shoulder peaks circled with dash line are observed besides characteristic peaks of the PEDOT as shown in Fig. 2, and the shoulders can be indexed to the well-documented G and D bands of GO nanosheets, respectively, which suggests that GO nanosheets have been successfully incorporated into the PEDOT film. It is known that G band reveals the E_2g_ phonon of C sp^2^. After the hybridization, a G band shift (from 1602 cm^−1^ to 1609 cm^−1^) was observed, which can be ascribed to π–π stacking interaction between the PEDOT nanoparticles and GO nanosheets atoms.^28^

### 3.3 XPS spectra

**Fig. 3:**
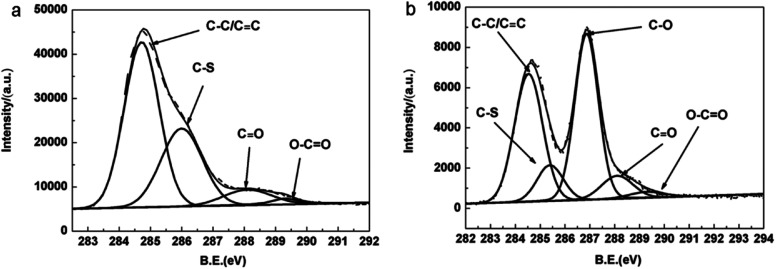
C 1s XPS spectra of (a) PEDOT film, (b) PEDOT–GO film.

X-ray photoelectron spectroscopy was carried out to investigate chemical and electronic structure of the samples. Fig. 3(a) and (b) show C 1s core level spectra of the pristine PEDOT film and PEDOT/GO hybrid nanostructures, respectively. As shown in Fig. 3(a), the strong peak centered at 284.7 eV for the PEDOT film can be attributed to the C–C and C=C bond. It is clear to differentiate the C–S peak at 286.3 eV, and small peaks at 288.1 eV and 289.3 eV which originate from C=O and O–C=O groups, respectively.^29,30^ In terms of C 1s XPS spectra of the PEDOT/GO hybrids, a rather strong peak at 286.9 eV is observed besides characteristic peaks of PEDOT as shown in Fig. 3(b), which can be assigned to C–O (epoxy and hydroxyl) bond.^29–31^ It is widely recognized that abundant epoxy groups and hydroxyl on the GO sheets surface make the GO surface negatively charged, which allows the *in situ* electro-polymerization of EDOT and favors the formation of uniform PEDOT nanoparticles on the GO nanosheets surface.

### 3.4 Electrochromic performance

**Fig. 4:**
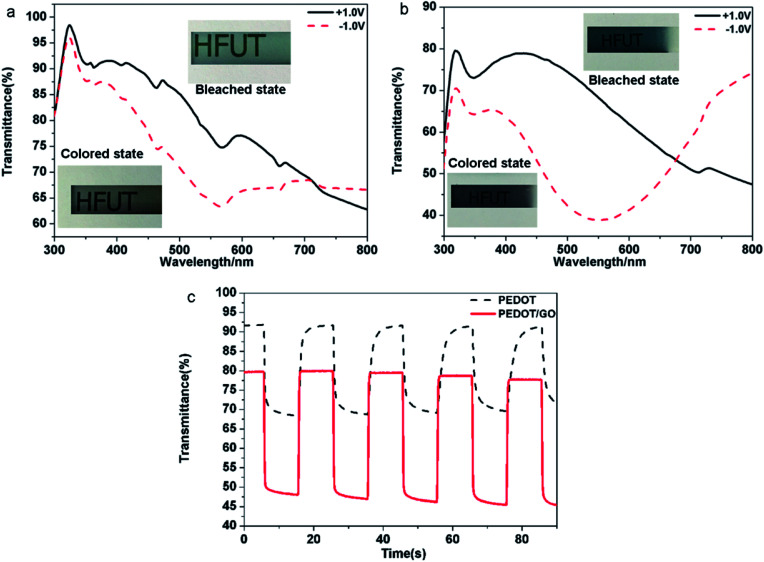
Transmittance spectra of (a) PEDOT film and (b) PEDOT–GO film recorded under different dc potentials varying from −1.0 V to +1.0 V in the ionic liquid: 1 M LiClO_4_/PC solution, (c) dynamic optical transmittance of PEDOT and PEDOT–GO film under square wave potential oscillating between +1.0 V and −1.0 V at a time step of 10 s recorded at 480 nm.

**Fig. 5:**
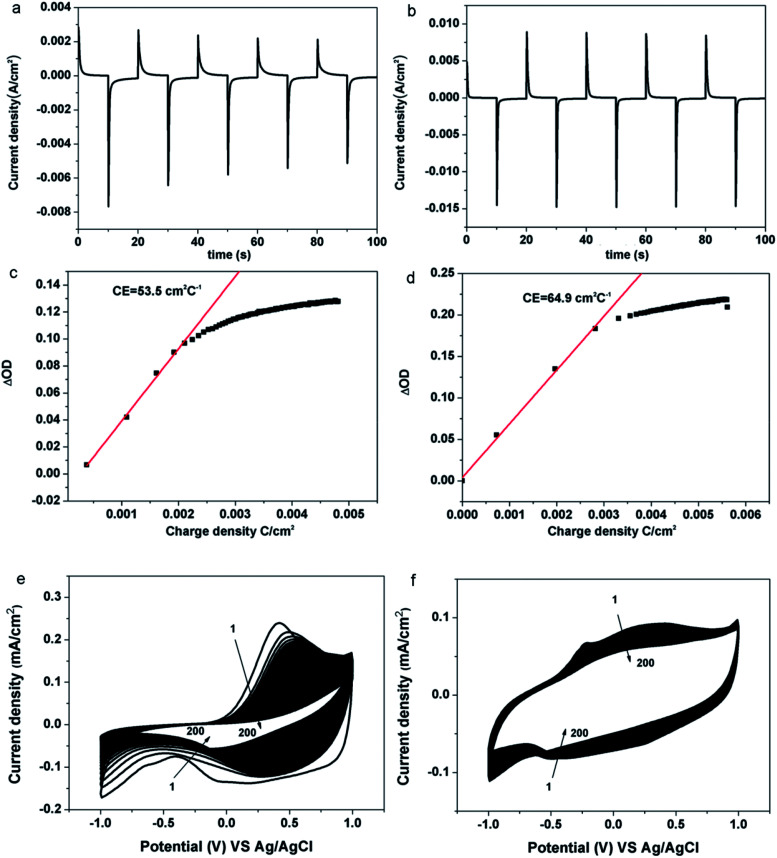
Corresponding chronoamperometry curves for the (a) PEDOT film and (b) PEDOT/GO film, variation of the *in situ* optical density (OD) *vs.* charge density for (c) PEDOT film and (d) PEDOT–GO film, cyclic voltammograms of the (e) PEDOT film and (f) PEDOT–GO film for 200 cycles.

Electrochromic performance of the samples was evaluated in a three-electrode electrochemical cell using 1 M LiClO_4_/PC solution as an electrolyte by combining the UV-VIS-NIR spectrophotometer and the electrochemical workstation. The area of the film is 1 cm^2^. (Details of constructing the device and measurement procedure can be found in the Experimental section). As shown in Fig. 4, typical transmittance spectra of the pristine PEDOT and the PEDOT/GO hybrids nanostructures were recorded under different potential varying from −1.0 V (*vs.* Ag/AgCl) to +1.0 V. For the pristine PEDOT film (Fig. 4(a)), color of the film changed from blue to purple at −1.0 V, and a distinct peak at 560 nm corresponds to a π–π* transition which suggests the formation of reduced polymer and the neutralization of bipolarons by electrons.^32^ During the oxidation of the PEDOT film by adjusting the potential to +1.0 V, the transmittance peak due to the π–π* transition still remained, implying that the doping level was not high and the new polarization level was not completely established. In terms of the PEDOT/GO hybrid nanostructures, the optical contrast from 300 nm to 800 nm is obviously higher than that of the pristine PEDOT film as shown in Fig. 4(b). The transmission edge of the π–π* transition peak for the PEDOT/GO hybrid nanostructures exhibits a blue shift with respect to that of the pristine PEDOT film, suggesting narrower band gap of the PEDOT film as compared to the PEDOT/GO film. As shown in Fig. 4(c), dynamic contrast between the reduced and oxidized states of the PEDOT/GO hybrid nanostructures is about 31.4% at the wavelength of 480 nm, which is evidently higher than 23.4% of the pristine PEDOT film. The coloring time (300 ms) and bleaching time (400 ms) of the PEDOT/GO hybrid nanostructures are much shorter than those of the PEDOT film (coloring time: 1500 ms, bleaching time: 1800 ms). The morphology and structure characterization demonstrate that the hybrid nanostructures benefit the charge transfer process and favor deeper ion injection into the active layer which evidently promote the optical contrast and response speed of the electrochromic electrode. It should be noted that the bleaching time is longer than the coloring time for both of the samples due to low doping degree of the conductive polymer as well as decreasing electrical conductivity under the negative potential, which impedes the electron transportation. Compared to reported layer-by-layer grown poly-(hexyl viologen)–PEDOT/PSS,^33^ PEDOT–RGO nanocomposite and PEDOT–ILFG nanocomposite,^32^ our hybrid has exhibited quicker coloring and bleaching time as well as higher optical contrast. As shown in Fig. 5(a), peak value of cathodic or anodic current density for the pristine PEDOT film shows irregular changes while the PEDOT/GO hybrid nanostructures exhibit identical peak value current density as shown in Fig. 5(b), indicating obviously better cyclic stability of the PEDOT/GO nanostructures than the pristine PEDOT film. Coloring efficiency is another important criterion evaluating electrochromic materials which is defined as the production of maximum possible change in color by the least amount of injected charges. In order to calculate this value, the change in the optical density (ΔOD) is supposed to be divided by corresponding charge density (corresponding injected/ejected charge density (*Q*) per unit area), the calculation formulas of which are shown as follows:^34,35^CE = ΔOD/*Q*ΔOD = log(*T*_b_/*T*_c_)where CE (cm^2^ C^−1^) is the coloring efficiency at a given wavelength. Coloration efficiencies of the pristine PEDOT and PEDOT/GO hybrid nanostructures were obtained from ΔOD *versus* injected charge density plots at the wavelength of 480 nm (Fig. 5(c and d)). The CE value of the PEDOT/GO hybrid nanostructures is figured out to be 64.9 cm^2^ C^−1^ which is 21.3% higher than the 53.5 cm^2^ C^−1^ of the pristine PEDOT film. It indicates that the PEDOT/GO hybrid nanostructures are able to more efficiently utilize the intercalated charge than the PEDOT film, translating a greater number of electrochemically addressable sites into higher coloring efficiency. The electrochemical behavior comparison of the two films as well as the contribution of GO nanosheets to the circulatory stability was evaluated by cyclic voltammetry tests in an ionic liquid of 1 M LiClO_4_/PC solution as shown in Fig. 5(e) and (f), respectively. In the first circle, an anodic peak appeared at about 0.38 V for the pristine PEDOT film due to the ClO_4_^−^ ions doping, and the film displayed blue color. In the reverse scan, a cathodic peak commenced at about −0.07 V due to anion extraction, and the film presented purple color. As for the PEDOT/GO hybrid film, the oxidation peak and reduction peak present at −0.22 V and −0.40 V respectively, and the film exhibited dark blue (reduction state) and light blue (oxidation state). These phenomena can be explained by anion extraction and insertion and the equations clarifying the redox process of both PEDOT film and PEDOT/GO film are shown as follows. The cycling stability of (e) PEDOT film and (f) PEDOT/GO hybrid film was studied by consecutive cyclic voltammetry to 200 cycles.

There was only a slight decrease in the current for the PEODT/GO hybrid film while the current decline was large for the pristine PEDOT film. The anodic peak current and cathodic peak current dropped for PEODT/GO hybrid film by 42% and 17%, respectively, while the anodic peak current and cathodic peak current for PEDOT film dropped by 91% and 71%, respectively. With increasing cycles, shifts were observed for the oxidation peak and reduction peak of the pristine PEDOT nanostructures indicating irreversible changes in the nanostructure caused by peroxidion and over-reduction. The results indicate that the PEDOT/GO hybrid film shows superior circulatory stability and has a much better electrochemical stability than the pristine PEDOT film. It can be proposed that the GO nanosheets act not only as a functionalized substrate for the uniform growth of the PEDOT nanoparticles, but also as a buffer layer providing sufficient space for the volume change of the PEDOT during the redox process, which guarantees high cycle stability.
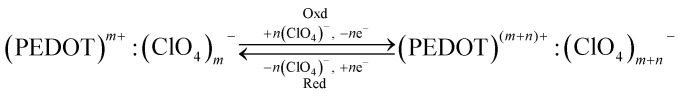

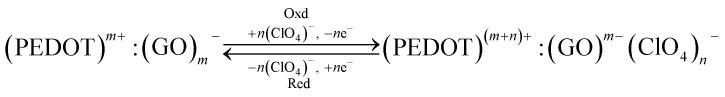


## 4 Conclusions

PEDOT nanoparticles/graphene oxide nanosheets hybrid nanostructures have been obtained by an *in situ* electro-polymerization technique. The hybrid nanostructures exhibit much shorter response time (300 ms for coloring and 400 ms for bleaching) than the pristine PEDOT nanostructured film (1800 ms for coloring and 1500 ms for bleaching). And the coloring efficiency is increased by 21.3% from 53.5 cm^2^ C^−1^ before hybridization to 64.9 cm^2^ C^−1^ after hybridization. Optical contrast was increased by 34.2% from 23.4% before hybridization to 31.4% after hybridization. In general, the ECDs achieved in this work have features of superfast response, superior optical contrast and improved cyclic stability, which can be applied in the smart window, smart displays, electronic papers and many other fields that require quick response. In addition, the *in situ* electro-polymerization method employed in this work can be extended to hybridize graphene oxide with other conductive polymer materials for electrochromic applications.

## Conflicts of interest

There are no conflicts of interest to declare.

## Supplementary Material

## References

[cit1] Lu C.-H., Hon M.-H., Kuan C.-Y., Leu I.-C. (2016). RSC Adv..

[cit2] Gazotti W. A., Paoli M. A. D., Casalbore-Miceli G., Geri A., Zotti G. (1999). J. Appl. Electrochem..

[cit3] Argun A. A., Cirpan A., Reynolds J. R. (2003). Adv. Mater..

[cit4] Delongchamp D., Hammond P. T. (2001). Adv. Mater..

[cit5] Heo J., Oh J.-W., Ahn H.-I., Lee S.-B., Cho S.-E., Kim M.-R., Lee J.-K., Kim N. (2010). Synth. Met..

[cit6] Tallman D. E., Spinks G., Dominis A., Wallace G. G. (2001). J. Solid State Electrochem..

[cit7] Gunbas G., Toppare L. (2012). Chem. Commun..

[cit8] Liu S., Xu L., Li F., Xu B., Sun Z. (2011). J. Mater. Chem..

[cit9] Augusto T., Teixeira Neto É., Teixeira Neto Â. A., Vichessi R., Vidotti M., de Torresi S. I. C. (2013). Sol. Energy Mater. Sol. Cells.

[cit10] Kateb M., Safarian S., Kolahdouz M., Fathipour M., Ahamdi V. (2016). Sol. Energy Mater. Sol. Cells.

[cit11] Cho S. I., Hwa Choi D., Sangho Kim A., Bok Lee S. (2005). Chem. Mater..

[cit12] Cho S. I., Xiao R., Bok Lee S. (2007). Nanotechnology.

[cit13] Ortiz D. N., Pinto N. J. (2017). Thin Solid Films.

[cit14] Omar V., Freddy W., Eduardo V., Jeileen L., Stephanie R., Nicholas J. P., Luis R. (2017). Polym. Sci..

[cit15] Krishnamoorthy K., Ambade A. V., Kanungo M., Contractor A. Q., Kumar A. (2001). J. Mater. Chem..

[cit16] Zhen S., Xu J., Lu B., Zhang S., Li Z., Li J. (2014). Electrochim. Acta.

[cit17] Cai G. F., Tu J. P., Zhou D., Zhang J. H., Wang X. L., Gu C. D. (2014). Sol. Energy Mater. Sol. Cells.

[cit18] Belekoukia M., Ramasamy M. S., Yang S., Feng X., Paterakis G., Dracopoulos V., Galiotis C., Lianos P. (2016). Electrochim. Acta.

[cit19] Chee W. K., Lim H. N., Huang N. M., Harrison I. (2015). RSC Adv..

[cit20] Hirata M., Gotou T., Horiuchi S., Fujiwara M., Ohba M. (2004). Carbon.

[cit21] Li D., Muller M. B., Gilje S., Kaner R. B., Wallace G. G. (2008). Nat. Nanotechnol..

[cit22] Österholm A., Lindfors T., Kauppila J., Damlin P., Kvarnström C. (2012). Electrochim. Acta.

[cit23] Lee S. H., Dreyer D. R., An J., Velamakanni A., Piner R. D., Park S., Zhu Y., Kim S. O., Bielawski C. W., Ruoff R. S. (2010). Macromol. Rapid Commun..

[cit24] Pu Z., Liu Q., Asiri A. M., Sun X. (2014). J. Appl. Electrochem..

[cit25] Liu S., Tian J., Wang L., Luo Y., Sun X. (2011). Analyst.

[cit26] Ramesha G. K., Sampath S. (2009). J. Phys. Chem. C.

[cit27] Garreau S., Louarn G., Buisson J. P., Froyer G., Lefrant S. (1999). Macromolecules.

[cit28] Park C., Yoo D., Im S., Kim S., Cho W., Ryu J., Kim J. H. (2017). RSC Adv..

[cit29] Bose S., Kuila T., Uddin M. E., Kim N. H., Lau A. K. T., Lee J. H. (2010). Polymer.

[cit30] Szabó T., Berkesi O., Forgó P., Josepovits K., Sanakis Y., Petridis D., Dékány I. (2006). Chem. Mater..

[cit31] Zhang J., Yang H., Shen G., Cheng P., Zhang J., Guo S. (2010). Chem. Commun..

[cit32] Saxena A. P., Deepa M., Joshi A. G., Bhandari S., Srivastava A. K. (2011). ACS Appl. Mater. Interfaces.

[cit33] Delongchamp D. M., Mark Kastantin A., Hammond P. T. (2003). Chem. Mater..

[cit34] Granqvist C. G., Green S., Niklasson G. A., Mlyuka N. R., von Kræmer S., Georén P. (2010). Thin Solid Films.

[cit35] Chen L. C., Ho K. C. (2001). Electrochim. Acta.

